# Translating Alzheimer’s Disease Mechanisms into Therapeutic Opportunities

**DOI:** 10.3390/biom15091290

**Published:** 2025-09-08

**Authors:** Jiejia Li, Liyun Wang, Xiaodan Zhang, Jianhua Shi, Yizhun Zhu, Han Wang, Xiangyang Zhu, Qing Zhu, Jia-Lie Luo

**Affiliations:** 1 School of Pharmacy, Nantong University, No. 9, Seyuan Road, Nantong 226001, China; 2 Institute for Translational Neuroscience, Second Affiliated Hospital, Nantong University, Nantong 226001, China; 3 Centre for Neural Developmental and Degenerative Research, Nantong University, Nantong 226001, China; 4 Laboratory of Drug Discovery from Natural Resources and Industrialization, Macau University of Science and Technology, Macau, China; 5 School of Pharmacy, Macau University of Science and Technology, Macau, China; 6 Department for Neurology, Second Affiliated Hospital, Nantong University, Nantong 226001, China

**Keywords:** Alzheimer’s disease, pathogenesis, biomarker, therapeutic strategies

## Abstract

Alzheimer’s disease (AD), characterized by progressive cognitive decline and functional impairment, is the most prevalent cause of dementia, and it poses a significant socioeconomic and caregiving burden on patients, families, and healthcare systems. Notwithstanding comprehensive research, the precise causes underlying AD remain ambiguous. Evidence increasingly indicates that AD is a multifactorial and heterogeneous disease involving a mix of genetic and environmental factors. The amyloid-cascade hypothesis, neuroinflammation and immunity, vascular pathology, and oxidative stress all fulfill significant functions in the onset and development of AD. This review primarily examines the critical pathogenesis, key biomarkers, and novel therapeutic strategies of Alzheimer’s disease to inform future research directions.

## 1. Introduction

Alzheimer’s disease (AD) is a gradual neurological condition marked by memory impairment and deterioration of cognitive abilities that can affect behaviors, language, and problem-solving and gradually lead to patients becoming incapable of carrying out everyday tasks on their own. According to a multicenter study, the duration of AD is about 20 years in total, including mild cognitive impairment (MCI), the preclinical stage, and the dementia stage [1]. Patients live for another 8 to 10 years after clinical diagnosis until death [2]. The majority of AD cases are sporadic (sAD), while only a small proportion are familial (fAD), typically associated with autosomal dominant genetic mutations [3]. Since Alois Alzheimer documented the initial case in 1907, scientists have studied AD for more than a century [4,5]. Unfortunately, the pathogenesis of AD is still unclear. The deposition of amyloid-beta (Aβ), typically regarded as the initial phase of the disease’s development, may occur 10–15 years prior to the onset of clinical symptoms, during which individuals remain cognitively intact. The increase in tau protein transpires thereafter, in proximity to the initiation of neurodegeneration [6,7]. Since AD deprives patients of their independence, it reduces their quality of life and places a substantial burden on families and society. There is an urgent need to develop strategies to identify and address the prolonged preclinical phase of Alzheimer’s disease. Early recognition of this phase is essential to enable the shift from treatment to prevention, aiming to delay or halt the disease before symptoms emerge. By targeting the underlying pathology earlier, therapeutic interventions could focus on slowing or preventing progression rather than solely managing symptoms, thereby improving long-term outcomes and potentially reducing the overall impact of Alzheimer’s disease.

## 2. Pathogenesis of AD

**Figure 1:**
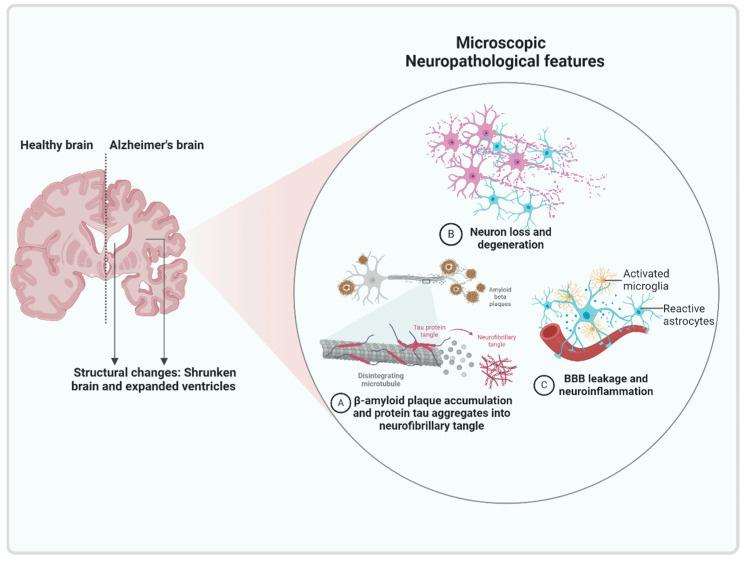
Histopathological Features of Alzheimer’s disease. Alzheimer’s brain is characterized by amyloid plaque accumulation, neurofibrillary tangles formed by abnormally hyperphosphorylated tau protein, neuronal loss resulting from gradual deterioration of neurons, vascular injury, and neuroinflammation.

In the last few decades, a breakthrough has been made in understanding AD, but the precise pathophysiology of AD remains a topic of ongoing debate. Genetic factors, amyloid senile plaques, hyperphosphorylated tau protein, oxidative stress, innate immune system disorder, and inflammatory responses are pivotal factors and participates in the pathogenesis of AD (Figure 1).

### 2.1. Genetic Factors

The mutations in three genes, namely amyloid precursor protein (APP), presenilin 1 (PSEN1), and presenilin 2 (PSEN2), are critical in the rare instances of fAD, which make the onset between 30–50 years of age, significantly earlier than sporadic cases [3]. For most sporadic cases, a complex interaction between genes and the environment is deemed to be the primary reason for ‘typical’ late onset sAD. It was reported that more than 40 risk alleles were identified with the large genome-wide association studies (GWAS) [8]. Among of them, APOE4 is the major genetic risk factor for Alzheimer’s disease. The APOE gene comes in three forms or variants (ε2, ε3, and ε4). APOEε4 is linked to an estimated 3- to 4-fold increased risk of AD and exerts multiple effects on AD. It disrupts the clearance of Aβ from the brain and is the most damaging isoform, capable of inducing neuropathology via several cellular mechanisms [9]. Moreover, the vascular anomalies in APOEε4-deficient and APOEε4-overexpressing animals predate neural impairment and may instigate neurodegenerative alterations through the cyclophilin A signaling pathway in the pericytes of cerebral blood arteries, which operates independently of Aβ [10]. SORL1, which contains domains from both the low-density lipoprotein receptor-like family and the vacuolar protein sorting-10 receptor family, decreases Aβ levels by binding to APP, thereby inhibiting its processing into Aβ, and by binding to Aβ to facilitate its transport to lysosomes for degradation. A significant fraction of the analyzed AD cases had high-risk SORL1 mutations (2%) [11]. In another study in Iceland, it was reported that an uncommon missense mutation (rs75932628-T) in the gene encoding the triggering receptor expressed on myeloid cells 2 (TREM2), resulting in an R47H substitution, was identified as a significant risk factor for AD [12]. Furthermore, *ABCA7* was identified as a risk gene for AD in GWAS. In a Belgian cohort, the intronic low-frequency variant rs78117248 exhibited the most robust connection with AD and retained significance after adjusting for the top GWAS single-nucleotide polymorphisms, rs3764650, rs4147929, and rs3752246 [13]. Besides that, the functional annotation of these risk loci indicates that neuroinflammatory (complement receptor 1, CR1) cholesterol and lipid metabolism dysregulation (complement regulators clusterin, CLU) [14], endosomal vesicle recycling pathways (BIN1, PICALM, CD2AP, and EPHA1), and others (PLD3) contribute to the pathogenesis of AD. Each of these genes contributes a minimal increase in risk; yet, when aggregated into a polygenic risk score, they can nearly double the predictive accuracy compared to random chance [15].

However, carriers of the APOE ε2 allele have a 2-fold lower lifetime risk of AD than non-carriers, which means a low likelihood of AD for homozygous APOEε2 allele carriers [16,17,18]. Apart from the APOE ε2 allele, the rare Ala673Thr Icelandic protective mutation of APP and a rare Pro522Arg amino acid change in the PLCG2 gene were linked to sustained cognitive health and a decreased risk of AD [19,20].

These genetic factors substantially influence susceptibility to AD, but they do not act in isolation. Emerging evidence suggests that gene–environment interactions critically shape disease onset and progression. Environmental influences including air pollution, sleep disruption, and chronic stress also interact with genetic predispositions to accelerate amyloid deposition, tau pathology, and neuroinflammation. For example, diet fats interact with APOE to modulate cognitive decline and APOE ε4 carriers who followed a Western (less healthy) diet experienced increased dementia risk [21,22].

### 2.2. Amyloid-Cascade Hypothesis

The cleavage of APP in the CNS occurs via two distinct pathways: the non-amyloidogenic pathway and the amyloidogenic pathway. The latter was considered to produce more extracellular Aβ through the sequential enzymatic actions of beta-site amyloid precursor protein–cleaving enzyme 1 (BACE-1), a β-secretase, and γ-secretase [23]. It is believed that imbalance of Aβ production and clearance (overproduction or reduced clearance) may induce the accumulation of pathological forms of Aβ, which may serve as the initiating factor in AD. Among the various Aβ peptides, Aβ40 and particularly Aβ42 exhibit a higher propensity for self-aggregation and fibril formation, ultimately leading to the development of plaques observable in pathological specimens [23]. PSEN1 and PSEN2 encode presenilin-1 and presenilin-2 proteins that are critical components of the γ-secretase complex, which cleaves APP [24].

The amyloid-cascade hypothesis posits that both soluble Aβ and amyloid plaques exhibit toxicity. They initiate the onset of AD through secondary damage or by initiating downstream events, including the hyperphosphorylation of tau protein, inflammation, oxidative stress, and excitotoxicity. In turn, the downstream events lead to neuronal cell death and a number of other neurotransmitter defects associated with AD, especially acetylcholine [25,26,27,28]. In other words, Aβ is a pivotal factor in the disease development, at least in fAD.

### 2.3. Tau Pathology

Tau is essential in numerous biological processes, including microtubule binding, axonal transport, modulation of signaling pathways, and adult neurogenesis [29]. Abnormally hyperphosphorylated and aggregated forms of tau are associated with neurofibrillary tangles (NFTs), which manifest in AD and other neurodegenerative conditions classified as tauopathies [30]. As the major component of the tangles, tau is also a prerequisite for the diagnosis of AD. It can act in parallel pathways with Aβ to cause AD and enhance each other’s toxicity, or act separately of Aβ to induce neurodegeneration [31,32]. The amyloid hypothesis posits that pathological changes in tau are considered to be subsequent events following Aβ deposition, paralleling with neuronal and synapse loss, and therefore the clinical symptoms and degree of AD are more closely linked to tau pathology.

Researchers demonstrated that tau can behave like prions, which shift stochastic refolding protein into a misfolded state by means of infection [33], and the latter infectious particle induces additional endogenous self-propagation of the misfolded proteins as seeds. Recent evidence endorses a concept for the intercellular transmission of proteinaceous pathogenic tau seeds. Abnormal tau seeds recruit and template endogenous tau proteins to form fibrils, leading to the propagation of tau pathology throughout various parts of the brain. The novel hypothesis of tau propagation provides insight into how AD progresses in a networked manner across brain regions. Understanding this process offers potential targets for therapies aimed at halting tau propagation and slowing the disease progression of AD and other neurodegenerative diseases [34].

### 2.4. Neuroinflammation and Immunity

Neurons have long been the primary focus in the study of age-related neurodegenerative diseases, such as AD and PD, due to the prevailing neuron-centric perspective. On the other hand, GWAS frequently found SNPs on TREM2, CD33, and other genes that are expressed on microglia and myeloid cells to be AD risk factors [35,36]. Microglia cells are innate immune cells that reside within the central nervous system (CNS) and are always active. Even in a “resting” condition, they continuously monitor the brain’s microenvironment by monitoring the parenchyma for potential dangers or changes [37]. In human AD brains, microglia are found to accumulate around amyloid plaques, suggesting that microglia may play a role in responding to amyloid-beta deposits [38]. Changes related to microglia were initially thought to be secondary responses to neurodegeneration in AD. However, recent research suggests that these microglial alterations play a vital part in the disease’s initiation and advancement. Microglia are now recognized as critical mediators of AD risk, contributing to inflammation, amyloid plaque clearance, and neuronal damage, which can influence the disease’s trajectory [39]. The involvement of microglia in the pathogenesis of AD is multifaceted. On one hand, they are responsible for engulfing and degrading Aβ plaques, thereby helping to clear toxic protein aggregates from the brain. Excessive microglial activation results in the release of inflammatory mediators, which promotes neurotoxicity and worsens neuronal damage. Furthermore, microglial function deteriorates progressively in an Aβ-dependent manner, as demonstrated by a decline in their phagocytic capacity in AD mouse models. Inflammatory cytokines can cause persistent microglial damage, suggesting that AD pathology will enter a vicious cycle [40,41].

**Figure 2:**
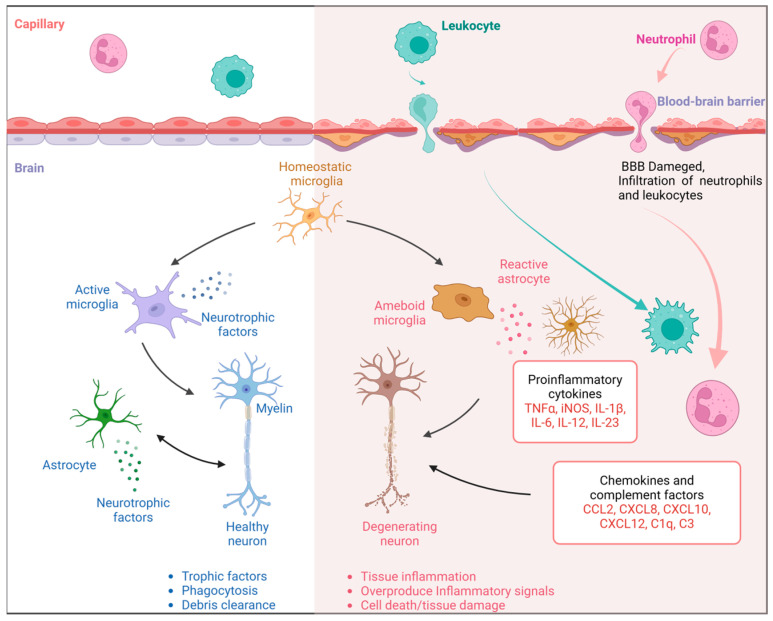
Inflammation in the Alzheimer’s brain. Distinct morphological features and functions of the vasculature, leukocytes, microglia, astrocytes, and inflammatory cytokines in healthy individuals and AD patients.

Another phenotype of AD is reactive astrogliosis, originally noticed by Alois Alzheimer himself, but to date, its contribution to AD remains an open question. Astrocytes serve an important role in brain stability, and their malfunction is now recognized as a major contributor to AD pathogenesis. In AD, astrocytes exhibit significant alterations in several signaling pathways, including glutamatergic and GABAergic signaling, potassium buffering, and calcium signaling [42]. In addition, hyperactive neuroinflammatory microglia cause a certain type of reactive astrocytes called A1 astrocytes. These astrocytes exhibit a significant reduction in their usual supportive functions, such as synaptic maintenance and neuroprotection, but instead acquire a neurotoxic phenotype releasing harmful factors that contribute to neuronal dysfunction and cell death, exacerbating neurodegeneration in AD [43]. Activated astroglia release acute-phase reactants, including alpha1-antichymotrypsin, alpha2-macroglobulin, and C-reactive protein, which may both exacerbate and alleviate Alzheimer’s disease (Figure 2).

### 2.5. Vascular Pathology

Apart from the aforementioned key hypotheses, vascular pathology in AD is a crucial factor in its initiation and development, linking cerebrovascular health closely to neurodegeneration. This pathology includes a range of issues, such as reduced cerebral blood flow, blood–brain barrier (BBB) breakdown, and small vessel disease, all of the above contribute to brain damage and cognitive loss in AD involving amyloid toxicity, oxidative stress, and ApoE genotype [44]. Ischemic disease impacts 60 to 90% of individuals with AD; in turn, one third of vascular dementia has coincidental pathological features of AD [45]. Another concept posits that vascular pathology compromises BBB integrity, leading to dementia irrespective of Aβ and tau pathology, particularly in carriers of the APOE ε4 allele [46,47].

A recent genome-wide gene study analyzing the interaction of cardiovascular and cerebrovascular risk factors (CVRFs) identified a key gene, FMNL2, whose expression is evident in the brains of individuals with cerebral infarcts and AD pathology and is correlated with amyloid and phosphorylated tau accumulation. Silencing of the fmnl2a gene inhibited gliovascular remodeling, decreased microglial activity, and exacerbated amyloidosis [48]. This study provides direct evidence that astroglial–vascular mechanisms contribute to the clearance of amyloid and tau, playing pivotal roles in the pathogenic process of AD. Chen et al. pointed out that high serum sodium levels, an indicator of the high cardiovascular risk, were associated with increased AD pathology, reduced hippocampal volume, and greater cognitive decline, suggesting that serum sodium could play a crucial role in AD progression via regulating vascular function [49]. A proteomics study using brain microvessels from the parietal cortex gray matter of AD patients and age-matched controls found 168 proteins with increased abundance in AD that are involved in cellular detoxification and antioxidative responses. These results indicate that the brain microvessels in AD are under elevated stress, driven by increased energy demands. In response, they activate compensatory mechanisms to counteract the ongoing oxidative and cellular damage characteristic of AD [50]. Single cell RNA sequencing data also provide evidence linking vascular defects to neurodegenerative diseases such as AD through gene expression profiles and pathways related to vascular function and neurodegeneration [51].

### 2.6. Oxidative Stress

In an AD brain, dysfunctional mitochondria release oxidizing free radicals, which cause considerable oxidative stress. It has been reported that oxidative stress occurs before the production of Aβ [52]. Aβ serves as a significant source of reactive oxygen species and reactive nitrogen species, thereby acting as a key initiator of oxidative stress.

It has been confirmed that several markers of oxidative damage were detected in AD patients. For example, the 3-nitrotyrosine residue (a production of ROS) concentration in the CSF is negatively linked to the Mini-Mental State Examination score [53]. The levels of MDA and 4-HNE increased in AD brains [54]. Isoprostane (IsoPs), as the product of lipid peroxidation, shows an increased level in the CSF in AD [55].

## 3. Biomarkers of AD

Biomarkers for AD are quantifiable signs that facilitate the detection, diagnosis, and monitoring of disease progression as well as the assessment of therapy responses, including amyloid-beta plaque buildup, tau protein tangles, neurodegeneration, and neuroinflammation. These biomarkers are detectable through imaging techniques, cerebrospinal fluid (CSF) analysis, and, increasingly, blood tests, enabling a less invasive approach to diagnosis. AD biomarkers enable the detection of the disease in its earliest stages, thereby allowing for early intervention and more effective management. This makes them valuable because changes in the neurochemistry and anatomy of the brain usually occur decades before people experience obvious cognitive symptoms [49]. By enabling more precise diagnosis and personalized treatment approaches, biomarkers are pivotal in advancing our understanding of AD and improving patient outcomes.

### 3.1. CSF Biomarkers

CSF is in proximity to the CNS and is usually considered a valid representative of the CNS. Core CSF biomarkers for AD include levels of Aβ_42_, total tau (t-tau), and phosphorylated tau (p-tau) [56,57], which are the principal elements of amyloid plaques and tau tangles, the defining characteristics of AD. Aβ42 indicates cortical amyloid accumulation, t-tau signifies the degree of neurodegeneration, and p-tau is associated with neurofibrillary pathological alterations. In the MCI stage, all the three biomarkers exhibit good diagnosis accuracy, specificity, and susceptibility.

The deposition of senile plaques is regarded as an early occurrence in the progression of AD, occurring several years prior to the manifestation of clinical symptoms. Aβ is the most important component of senile plaques; therefore, it is widely recognized as a dependable biomarker reflecting the pathophysiological processes of AD. Interestingly, Aβ has also been reported in the CSF of healthy persons, although its concentration is diminished in AD. The decline is attributable to the consumption of Aβ resulting from its aggregation in the brain, resulting in a growth of senile plaques. Therefore the ratio of Aβ_42_ to Aβ_40_ is often used to increase the diagnostic accuracy. Aβ_42_, generated through the proteolytic cleavage of APP, is regarded as especially hazardous and likely to agglomerate. The modifications of Aβ_42_ in CSF usually occur in healthy individuals between 40 and 50 years of age, which is a long preclinical stage of the disease [58]. Tau protein is a structural protein, and hyperphosphorylated forms of tau are prone to aggregation to form NFTs. In the CSF of normal individuals, tau can be detected to some extent, but increased concentration of tau, particularly p-tau, is more frequently detected in MCI and AD patients [59,60,61]. Therefore, scholars speculate that the existence of Aβ42 and p-tau biomarkers in the CSF of asymptomatic individuals indicates early pathological changes, suggesting a critical window for timely therapeutic intervention [58].

Recent advancements in AD biomarker research have expanded beyond the conventional CSF markers Aβ_42_, t-tau, and p-tau. According to a recent meta-analysis, neurofilament light protein (NFL) is an additional marker of neurodegeneration that has been assessed as a viable CSF biomarker. NFL was strongly associated with AD together with the fundamental indicators of AD. In addition, several other biomarkers are receiving increasing attention such as neurogranin, synaptosomal-associated protein-5(SNAP25), visinin-like protein 1(VILIP-1), and YKL-40 [62,63,64]. Among them, presynaptic proteins (e.g., SNAP25) and dendritic proteins (e.g., neurogranin) are elevated in the CSF in MCI and AD patients [65,66,67]. YKL-40 (chitinase-3-like-1, cartilage glycoprotein-39) is a secreted glycoprotein regarded as a possible biomarker for active inflammation and neuronal damage in several human disorders. CSF levels of YKL-40 elevate in neurodegenerative diseases, especially in preclinical AD and frontotemporal dementia (FTD) [68]. The astrocyte-specific protein, glial fibrillary acidic protein (GFAP), is a highly brain-specific protein, which has been observed to be elevated in the CSF of brain of AD, reflecting the reactive astrocytes within the cerebrum. The findings suggest that GFAP levels could be used to detect early-stage AD [69].

Ubiquitin has also been recognized as a potential marker involved in protein degradation and clearance. It has been detected with elevated levels in various neurodegenerative diseases, including AD and Parkinson’s disease (PD). Kandimalla et al. reported elevated levels of ubiquitin in the CSF of AD. It showed diagnostic performance comparable to other core biomarkers [70]. This change could also be detected by mass spectrometric analysis in the CSF of AD [71]. Inflammatory mediators, including IL-1β, TNF-α, and IL-6, have been quantified in the CSF as indicators of neuro-immunomodulatory alterations associated with AD pathogenesis; however, AD-related neuroinflammation involves an intricate web of immune cells, cytokines, and chemokines, making it challenging to pinpoint a single or consistent pattern that correlates with disease stage or severity. They cannot be considered as reliable biomarkers. Researchers also focused on neurotrophic factors in AD. For example, the level of BDNF has been observed to be elevated in the brain, CSF, and blood of AD patients [72].

### 3.2. Imaging Biomarkers

Another important category of biomarkers are imaging biomarkers. Imaging is essential in the clinical evaluation of patients with probable AD. The three most validated neuroimaging indicators for AD are medial temporal lobe atrophy identified via MRI, posterior cingulate and temporoparietal hypometabolism assessed using 18FDG-PET, and cortical Aβ accumulation visualized by amyloid-PET imaging. Compared to the visual scale ratings of medial temporal atrophy, hippocampal volumetry is generally considered more accurate for assessing neurodegeneration in AD, because it provides a quantitative measure of hippocampal volume instead of relying on subjective interpretation of brain imaging [73,74].

Amyloid-PET makes it possible to identify and validate fibrillar amyloid plaques in the cerebellum and serves as the most effective diagnostic instrument in the differential diagnosis of AD. Amyloid-PET imaging has profoundly influenced the clinical treatment of individuals with MCI and AD [75]. Three tracers including ^18^F-Florbetaben (Neuraceq), ^18^F-Florbetapir (Amyvid), and ^18^F-Flutemetamol (Vizamyl) were authorized by the FDA for imaging Aβ plaques in the brain using amyloid-PET [76]. Despite the clinical importance, their use is often limited because of the financial burden on patients and families, as the test is frequently not covered by health insurance. In addition, an abnormal scan is not conclusively diagnostic for AD because amyloid accumulation can also occur in cognitively normal older individuals. A negative scan is valuable because it effectively rules out AD as the cause of dementia symptoms, pointing instead to other forms of dementia [77,78]. In order to acquire much higher sensitivity, amyloid-PET should be measured in local regions, such as temporobasal and frontmedial areas at the earliest stages [79].

Tau-PET imaging serves as a significant indicator for the differential diagnosis of AD tauopathy compared to other neurodegenerative tauopathies. In addition, tau and synapse PET scans are only available on a research basis at present [80]. The spatial distribution (topography) of tau deposition detected by tau-PET correlates strongly with specific cognitive impairments and clinical phenotypes of AD. Different patterns of tau accumulation are linked to distinct cognitive symptoms, reflecting the heterogeneity of AD presentations [81,82,83]. In a word, the long-term study of tau-PET gives us a novel perspective to enhance understanding of tau’s role and its interaction with Aβ.

Identifying early indicators of capillary injury and BBB disruption has emerged as a novel and critical research focus in AD. These vascular changes often precede amyloid-beta (Aβ) and tau pathology, suggesting that they could serve as early indicators of disease onset and progression [47]. Biomarkers for vascular dysfunction and neuronal damage in both blood and CSF may be used to prevent AD in the forthcoming period, but this is still a long way off [84,85,86]. It has been reported that there is a decrease of synaptic vesicle glycoprotein 2A (SV2A) interaction in the hippocampus in patients with MCI or AD. Therefore, the development of PET imaging ligands targeting SV2A will open new avenues to explore brain synaptic density [80,87]. Additional PET markers targeting α-synuclein, neurotransmitter systems, TDP-43, and neuroinflammation are highly anticipated. The further development of techniques in hybrid PET-MRI, dual-phase amyloid-tau PET imaging, and multimodal neuroimaging has the potential to significantly improve the diagnostic capabilities for AD [79].

## 4. Progress of Treatments

In the past few decades, scientists have carried out intensive research on the pathogenesis of AD. Several treatment techniques have been suggested to tackle the fundamental causes of neurodegeneration, primarily concentrating on targeting the Aβ cascade to inhibit the production of harmful amyloid aggregates. However, other researchers suggested stopping focusing on amyloid-β as AD’s primary cause after the two well-publicized clinical trials of medications for the disorder ended in failure. Here, we review the current landscape of AD treatments, spanning preclinical and early clinical development (Table 1).

### 4.1. Symptomatic Treatment

Symptomatic treatments aim to alleviate cognitive and non-cognitive symptoms without altering the underlying disease progression, including cholinesterase inhibitors and N-methyl-D-aspartate (NMDA) receptor antagonists. The cholinesterase inhibitors, including donepezil, rivastigmine, and galantamine, are used as the first-line drugs for the symptomatic therapy of the dementia stage. This treatment strategy stems from autopsy findings that revealed significant loss of cholinergic neurons in early or moderate AD patients [88]. By enhancing acetylcholine availability through the inhibition of its degradation in the synaptic cleft, cholinesterase inhibitors can enhance the efficiency of deteriorating cholinergic neurons, moderately alleviate the signs and symptoms of disease, and support cognitive abilities and functionality in individuals with mild to moderate conditions. However, they do not modify the trajectory of illness or influence the fundamental pathophysiology. While these three drugs are generally equally effective in improving cognitive function and daily living activities, their dosing frequency, dose variation, and delivery methods differ, facilitating customized treatment according to the specific requirements of each patient [89]. Researchers indicated individuals with moderate to severe AD who maintained medication with donepezil demonstrated reduced impairment compared to those who ceased treatment [90].

Memantine is a low-affinity antagonist of the NMDA receptor. It can diminish L-glutamate excitatory neurotoxicity while preserving its normal physiological functions. Consequently, it serves as an alternate symptomatic therapy for mild to severe AD [91]. Memantine is considered a primary treatment for moderate to severe AD and is especially effective in managing behavioral disturbances. Either as an independent therapy or in conjunction with a cholinesterase inhibitor, memantine has shown potential benefits for individuals with MCI to severe dementia [90]. Memantine may cause side effects such as constipation and headache.

### 4.2. Target Aβ and Tau Directly

The production of Aβ is primarily driven by the stepwise cleavage of amyloid precursor protein (APP) by two key enzymes: β-secretase (BACE1) and γ-secretase. Targeting these secretases to inhibit or modulate their activity is a promising therapeutic tactic for reducing Aβ formation and potentially preventing cognitive deterioration. It has been recently shown that subchronic administration of γ-secretase inhibitors diminished cognitive function around three months in both Tg2576 and wide-type mice. On the other hand, γ-secretase modulators were effective in improving cognitive impairment in Tg2576 mice, indicating that they could be useful as applicants for AD treatment regimens [92]. Orally bioavailable BACE1 inhibitors have demonstrated efficacy in decreasing CSF Aβ levels in APP transgenic mice and non-transgenic beagle dogs. Unfortunately, because of the toxicity in healthy human volunteers, BACE1 has been prevented from moving to later-stage clinical trials [93].

Anti-Aβ aggregation drugs are also a concern of researchers. Yao et al. chose a fragment of Aβ peptide-binding alcohol dehydrogenase (ABAD) that bound amyloid to form the DP-TAT-Mito peptide (DP: decoy peptide; TAT: transduction of human immunodeficiency virus 1-transactivator; Mito: mitochondrial targeting peptide), which effectively inhibited ABAD-Aβ complex formation, oxidative stress, mitochondrial dysfunction, and enhanced cognitive function in AD mouse [94].

Lipoprotein receptor-related protein 1 (LRP1) is a transporter protein that transports Aβ out of the brain across the BBB. In vitro studies showed that increased levels of LRP1 may be associated with the active cannabinoid system to reduce the accumulation of intracellular Aβ and prevent proteotoxicity and inflammation [95,96].

### 4.3. Immunological Therapy

Researchers also focused on immunotherapy strategies, including active and passive immunization. Patients took part in a vaccine trial for full-length Aβ (AN1792) and were identified as reactors in the phase 2a study; they maintained low but noticeable levels of anti-AN1792 antibodies for 4.6 years. Nevertheless, this trial was prematurely stopped because of the incidence of severe meningoencephalitis in 6% of the 300 participants [97,98]. The Aβ vaccine has potential therapeutic effects when it is safe. CAD106, a novel active Aβ treatment, was created to stimulate antibody production without T-cell participation, thereby avoiding the harmful effects noted in earlier vaccine trials. This trial indicated that CAD106 exhibited a favorable safety profile and an acceptable antibody response in patients with AD over two years of follow-up. However, clinical trials with increased samples and multiple doses are necessary to further validate the safety and efficacy of CAD106 [99].

Tau protein is essential for maintaining the stability of neuronal microtubules. However, hyperphosphorylated and misfolded tau contributes to the pathophysiology of neurodegeneration of AD when it accumulates to form neurofibrillary tangles within neurons, displacing intracellular components. Therefore, inhibition or modulation of the aggregation and the spread of tau may be a potential therapy strategy. Chai et al. reported a passive immunotherapy approach targeting tau pathology using two mouse models, JNPL3 and P301S. They showed that peripheral delivery of two distinct anti-tau antibodies resulted in a decrease in biochemical indicators of tau pathology. Furthermore, in the P301S mice, this immunotherapy was able to delay the onset of functional decline, suggesting a potential therapeutic benefit. These findings highlight the promise of passive immunotherapy for tau as a strategy to mitigate tau-related neurodegeneration in AD and other tauopathies [100].

Bapineuzumab targets the N-terminus of Aβ. It could decrease the accumulation of amyloid and lower the CSF p-tau in APOE ε4 carriers in an 18-month trial, but unfortunately, it was not associated with any clinical benefit [101,102]. Solanezumab targets soluble Aβ and showed no benefit in cognitive clinical outcomes in a distinct phase 3 trial [103]. At the same time, researchers observed that other anti-Aβ antibodies showed harmful immunological responses following active immunization [97,104]. Nevertheless, the prospects for anti-Aβ antibody therapies are not entirely pessimistic. Researchers have created a humanized anti-Aβ monoclonal antibody, designated MABT, which may inhibit excessive microglial activation to mitigate harmful immunological responses. MABT can operate from various perspectives: It is constructed based on the IgG4 backbone, capable of preventing amyloid protein aggregation, breaking down already formed protofibrils, and blocking the cytotoxicity of Aβ to neurons and mixed cortical cell cultures. The most surprising fact was that the production of TNF-α was less in MABT-induced microglial. The safety of MABT has also been verified through clinical trials. Crinalezumab (MABT) has received funding for phase II/III prevention trials [105,106].

### 4.4. Target out of Aβ and Tau

As AD is a complex and multifactorial disease, traditional Chinese medicine (TCM) offers a unique approach to managing AD by leveraging the synergistic effects of its multi-ingredient formulations. Multiple studies have demonstrated the therapeutic benefits of TCM in modifying the disease progression and improving memory deficits. SuHeXiang Wan, a modified formulation of TCM, was reported to enhance memory function affected by Aβ and reduce Aβ levels and plaque accumulation in the cerebellum of AD mice [107]. Ginkgo biloba extract EGb 761^®^ (*Dr*. *Willmar Schwabe* GmbH & Co. KG karlsruhe, Germany) was demonstrated to have efficacy in modifying the cognitive behavior and neuropsychiatric symptoms of AD [108]. Salidroside had a protective effect through the inhibition of cytotoxicity and harm from oxidation caused by the buildup of Aβ in vitro [109,110]. Moreover, salidroside may enhance damaged hippocampal neurogenesis by eliminating reactive oxygen species in a streptozotocin-induced Alzheimer’s disease rat model [111].

Research indicates that antioxidants are helpful in mitigating aging and oxidative stress in AD models; nevertheless, clinical trials demonstrate that more thorough research is required to definitively prove these chemicals’ safety and effectiveness [112]. Curcumin, a renowned natural compound with significant anti-inflammatory and antioxidant properties, has been demonstrated to bind to plaques and diminish the development of Aβ oligomers and fibrils in a mice model [113,114]. Resveratrol is a natural anti-aging compound demonstrated to mitigate mitochondrial dysfunction in vitro [115]. The highest concentrations of antioxidants in mitochondria are MitoQ and SS31, which were denoted to enhance synaptic connections in neurons in an AD model [116]. Resveratrol, MitoQ, and SS31 are significant in modulating mitochondrial damage induced by elevated Aβ levels [117,118]. Vitamin E, a well-known antioxidant, is also of interest to researchers. A 16-week study found that when treated with vitamin E in combination with vitamin C and α-lipoic acid, there was no alteration in cerebrospinal fluid biomarkers related to AD, but a faster cognitive decline induced by lower levels of F2-isoprostane was shown [117]. Nevertheless, another study indicated that a certain dose of tocopherol mitigated cognitive deterioration in comparison to the placebo group [119]. These results suggest that further studies are needed to elucidate the exact effect and mechanism of vitamin E in AD treatment.

Previous findings indicated that S-propargyl cysteine (ZYZ-802), a novel hydrogen sulfide-modulated agent derived from garlic extract, enhances spatial learning and memory impairment caused by LPS-induced neuroinflammation by regulating the hydrogen sulfide pathway [120].

### 4.5. Non-Pharmaceutical Treatment

Alzheimer’s disease has been defined by dysfunction in the brain components and circuits that govern cognitive and memory abilities and activation of brain networks associated with memory retention, and acquisition may provide advantages. In a phase I study, patients received continuous deep brain stimulation (DBS) targeting the fornix and hypothalamus for 12 months. PET scans revealed a significant and early restoration of poor glucose utilization, and the Alzheimer’s Disease Assessment Scale cognitive subscale showed that cognitive decline could be slowed down in some patients [121].

Scanning ultrasound (SUS) is an innovative therapy that temporarily compromises the integrity of the BBB, enhancing local permeability and thereby facilitating the elimination and decrease of Aβ deposition in the cerebrum of AD patients. Researchers demonstrated that SUS was safe, exhibiting no edema, neurodegeneration, or ischemic damage, when applied to AD mice. The amount of plaque was lower in SUS-treated mice with AD compared to those that did not receive treatment; even more importantly, their ability to remember where things were, learn new locations, and recall information in the short term improved [122]. A recent study also reported that SUS could ameliorate memory deficits in in the APP23 mouse model of AD. However, it does not require the reduction of Aβ burden, indicating a different mechanism of action [123].

Studies have reported that exercise programs combining aerobic and resistance training, conducted at moderate intensity for a minimum of 45 min per session on multiple days each week, positively impact cognitive function in healthy older adults. Regular physical activity has been shown to enhance brain health by improving cerebral blood flow, reducing neuroinflammation, and promoting neuroplasticity. In terms of human physiology, moderate physical exercise can promote the increase of gray matter in the brain and reduce the risk of cognitive impairment [124,125,126].

Using an APP/PS1 transgenic mice model, it has been found that 20 weeks of treadmill exercise reduced hippocampal levels of NLRP3, IL-1β, and Aβ_1–42_ while attenuating microglial activation and alleviating neuronal damage [127]. The beneficial effects of exercise on AD have also been confirmed by systematic review and meta-analysis of randomized controlled trials, which showed that physical exercise intervention significantly improves cognitive performance in patients with AD [126,128,129]. Exercise is not only a supportive lifestyle intervention but also a potential disease-modifying strategy in AD. Its benefits span molecular, vascular, cognitive, and psychosocial domains, making it a cornerstone of non-pharmacological AD management and an important avenue for preventive and therapeutic research.

Dietary interventions are increasingly recognized as modifiable factors that may influence the onset and progression of Alzheimer’s disease (AD). Specific dietary patterns—such as the Mediterranean diet (MeDi), the DASH diet (Dietary Approaches to Stop Hypertension), and their hybrid, the MIND diet (Mediterranean-DASH Intervention for Neurodegenerative Delay)—have shown consistent associations with reduced risk of cognitive decline and AD [130,131]. Dietary gradients, such as omega-3 fatty acids (DHA/EPA), resveratrol and related compounds exhibiting antioxidant, anti-amyloid, anti-inflammatory, and neuroprotective effects, significantly lower dementia risk [132,133]. The effects of dietary modification have also been linked to microbiota, which has also been shown to contribute to neuroinflammation, Aβ accumulation, and cognitive decline in AD [134].

Research reveals that gut microbiota changes cause peripheral accumulation of chemicals including phenylalanine and isoleucine, which induce pro-inflammatory T helper 1 (Th1) differentiation and proliferation. These Th1 cells, in turn, promote M1 microglia activation, contributing to AD-associated neuroinflammation. GV-971, a sodium oligomannate, has emerged as a promising therapeutic agent targeting gut dysbiosis in AD. In a phase 3 clinical trial conducted in China, GV-971 demonstrated consistent cognitive improvement. Its mechanism of action involves suppressing gut dysbiosis, reducing phenylalanine and isoleucine deposition, modulating neuroinflammation, and ultimately ameliorating cognitive decline [135]. A recent experimental study using AD mouse models (APPPS1-21 and 5XFAD) revealed that GV-971 (sodium oligomannate) modulates gut microbiota, reduces amyloid pathology, attenuates reactive microglia and astrocytes, and alleviates neuroinflammation in a sex-dependent manner [136]. These results highlight the potential of gut microbiota-targeted therapies in AD treatment. A multidomain intensive lifestyle trial published in 2024 assessed participants with MCI or early dementia due to AD. Over 20 weeks, interventions targeting lifestyle (diet, exercise, and cognitive activity) improved measures of cognition [137]. Collectively, these studies indicate that non-pharmacological interventions—such as structured exercise programs, dietary patterns like the Mediterranean/MIND diets, cognitive stimulation, and microbiome-targeted approaches—serve as valuable adjuncts to pharmacological therapies for AD.

**Table 1:** Therapeutic strategies for AD.

References	Description	Drug’s Name	Category	
[88,89,90]	Enhance acetylcholine availability through the inhibition of its degradation in the synaptic cleft	Donepezil	Acetyl-cholinesterase inhibitors (AChEIs)	Symptomatic treatment
[88,89,90]		Galantamine		
[88,89,90]		Rivastigmine		
[90,91]	Reduce L-glutamate excitatory neurotoxicity	Memantine	N-methyl-D-aspartate receptor antagonist	
[92]	Restrain Aβ production	BACE1	β-secretase inhibitors	Target Aβ and Tau
[93]		LY450139	γ-secretase inhibitors	
[93]		GSM-2	γ-secretase modulator	
[94]	Anti-Aβ aggregation, mitigate mitochondrial dysfunction and enhance spatial learning and memory	ABAD	Aβ peptide-binding alcohol dehydrogenase	
[95,96]	Active cannabinoid system to mitigate the buildup of intracellular Aβ and avert proteotoxicity and inflammation	LRP1	Lipoprotein receptor-related protein	
[97,98]	Induce antibodies	AN1792	Aβ vaccine	Immunological therapy
[99]	Induce antibodies without T-cell to avoid the adverse events	CAD106		
[101,102]		Bapineuzumab	anti-Aβ antibodies	
[103]		Solanezumab		
[105,106]		Crenezumab		
[100]	Inhibit or modulate the aggregation and the spread of tau		anti-tau antibodies	
[107]	Enhance memory function affected by Aβ and reduce Aβ levels and plaque accumulation.	SuHeXiang Wan	Taditional Chinese Medicine	Target out of Aβ and Tau
[108]	Modulate cognitive behavior and neuropsychiatric symptoms	Ginkgo biloba extract EGb 761^®^		
[109,110,111,112]	Against cytotoxicity and oxidative damag	Salidroside		
[113,114]	Anti-inflammation and anti-oxidation	Curcumin		
[118]	Reduce mitochondrial dysfunction	Resveratrol		
[120]	Enhance spatial learning and memory impairments caused by LPS-induced neuroinflammation	S-propargyl cysteine		
[135,136]	Alterate gut microbiota composition, stimulate differentiation and proliferation of Th1 cells, improve cognition	GV-971	Intestinal microbiological regulator	
[121]	Reverse the impaired glucose utilization		Deep Brain Stimulation	Non-pharmaceutical treatment
[122,123]	Temporarily compromise the tight junctions of the BBB, increasing local permeability		Scanning ultrasound	
[124,125,126]			Aerobic and resistance training	

### 4.6. Limitations and Challenges

Despite decades of effort, most Aβ- and tau-targeted therapies have delivered limited clinical benefit. A major reason is that many interventions were applied too late in the disease course, when extensive neuronal loss had already occurred and could not be reversed. Moreover, several early agents targeted insoluble plaques rather than the more toxic soluble oligomeric species or failed to effectively engage pathogenic tau inside neurons. Clinical trials of BACE1 inhibitors even led to cognitive worsening due to off-target effects, and monoclonal antibodies have been constrained by amyloid-related imaging abnormalities (ARIA), particularly in APOE ε4 carriers. Importantly, Alzheimer’s disease involves multiple overlapping pathologies—including vascular dysfunction, immune dysregulation, and metabolic disturbances—that remain unaddressed by single-target amyloid or tau therapies, limiting their capacity to halt disease progression.

Future strategies are shifting toward earlier, biomarker-driven intervention and combination therapies. Next-generation antibodies are being engineered to selectively neutralize soluble oligomers, improve brain penetration, and reduce ARIA risk, with subcutaneous formulations under development to enhance accessibility. Other promising directions include therapies targeting multiple mechanisms and pathways such as TREM2 to restore protective immune responses, metabolic modulators like GLP-1 receptor agonists that improve insulin signaling and synaptic resilience, and agents aimed at vascular repair or gut–brain axis regulation, such as GV-971. Precision medicine approaches using plasma biomarkers (e.g., p-tau, Aβ42/40 ratio, NfL) now allow for earlier diagnosis, better patient selection, and adaptive treatment strategies. Together, these advances point toward multimodal and personalized interventions that go beyond protein clearance to address the broader pathophysiology of Alzheimer’s disease.

## 5. Concluding Remarks

The current review summarizes the potential mechanism of AD, including genetic factors, amyloid-cascade hypothesis, neuroinflammation and immunity, vascular pathology and oxidative stress, propose some related biomarkers from both CSF and imaging, and discuss therapeutic strategies including symptomatic treatment, immunological therapy, target in and out Aβ and Tau treatment, and non-pharmaceutical treatment. These therapeutic strategies can more or less mitigate the development of this dangerous disease. AD is currently untreatable, gaining a deeper understanding of its underlying mechanisms is crucial for advancing future treatments. Continued research into the pathophysiology, biomarkers, and therapeutic targets of AD holds promise for developing more effective interventions. It is hoped that this review will guide follow-up research efforts and inspire novel approaches to treatment, ultimately paving the way for breakthroughs that could slow, prevent, or even reverse the progression of this devastating neurodegenerative disorder.

## 6. Future Perspectives

Looking ahead, the future of Alzheimer’s disease research and therapy lies in integrating multimodal approaches that target the disease at multiple levels and at its earliest stages. The convergence of blood-based biomarkers, advanced neuroimaging, and genetic profiling will allow for earlier detection and precise patient stratification, enabling truly personalized medicine. Therapeutically, future strategies should move toward combination approaches that not only target amyloid and tau but also address neuroinflammation, vascular dysfunction, mitochondrial impairment, and gut–brain axis alterations, while integrating non-pharmacological interventions such as exercise, cognitive training, and dietary modulation, ultimately harmonizing these modalities for personalized treatment.

## Data Availability

Not applicable.

## References

[1] Vermunt L., Sikkes S.A.M., van den Hout A., Handels R., Bos I., van der Flier W.M., Kern S., Ousset P.J., Maruff P., Skoog I. (2019). Duration of preclinical, prodromal, and dementia stages of Alzheimer’s disease in relation to age, sex, and APOE genotype. Alzheimers Dement.

[2] Jack C.R., Knopman D.S., Jagust W.J., Petersen R.C., Weiner M.W., Aisen P.S., Shaw L.M., Vemuri P., Wiste H.J., Weigand S.D. (2013). Tracking pathophysiological processes in Alzheimer’s disease: An updated hypothetical model of dynamic biomarkers. Lancet Neurol..

[3] Bateman R.J., Aisen P.S., De Strooper B., Fox N.C., Lemere C.A., Ringman J.M., Salloway S., Sperling R.A., Windisch M., Xiong C. (2011). Autosomal-dominant Alzheimer’s disease: A review and proposal for the prevention of Alzheimer’s disease. Alzheimers Res. Ther..

[4] Morris R.G., Salmon D.P. (2007). The centennial of Alzheimer’s disease and the publication of “Uber eine eigenartige Erkankung der Hirnrinde” by Alois Alzheimer. Cortex.

[5] Zhang J., Kong G., Yang J., Pang L., Li X. (2025). Pathological mechanisms and treatment progression of Alzheimer’s disease. Eur. J. Med. Res..

[6] Hong X., Huang L., Lei F., Li T., Luo Y., Zeng M., Wang Z. (2025). The Role and Pathogenesis of Tau Protein in Alzheimer’s Disease. Biomolecules.

[7] Gao F., Hou Y., Wang Y., Liu L., Yi X., Xia N. (2025). Photothermal and Photodynamic Strategies for Diagnosis and Therapy of Alzheimer’s Disease by Modulating Amyloid-beta Aggregation. Biosensors.

[8] Jansen I.E., Savage J.E., Watanabe K., Bryois J., Williams D.M., Steinberg S., Sealock J., Karlsson I.K., Hagg S., Athanasiu L. (2019). Genome-wide meta-analysis identifies new loci and functional pathways influencing Alzheimer’s disease risk. Nat. Genet..

[9] Castellano J.M., Kim J., Stewart F.R., Jiang H., DeMattos R.B., Patterson B.W., Fagan A.M., Morris J.C., Mawuenyega K.G., Cruchaga C. (2011). Human apoE isoforms differentially regulate brain amyloid-beta peptide clearance. Sci. Transl. Med..

[10] Bell R.D., Winkler E.A., Singh I., Sagare A.P., Deane R., Wu Z., Holtzman D.M., Betsholtz C., Armulik A., Sallstrom J. (2012). Apolipoprotein E controls cerebrovascular integrity via cyclophilin A. Nature.

[11] Holstege H., van der Lee S.J., Hulsman M., Wong T.H., van Rooij J.G., Weiss M., Louwersheimer E., Wolters F.J., Amin N., Uitterlinden A.G. (2017). Characterization of pathogenic SORL1 genetic variants for association with Alzheimer’s disease: A clinical interpretation strategy. Eur. J. Hum. Genet..

[12] Jonsson T., Stefansson H., Steinberg S., Jonsdottir I., Jonsson P.V., Snaedal J., Bjornsson S., Huttenlocher J., Levey A.I., Lah J.J. (2013). Variant of TREM2 associated with the risk of Alzheimer’s disease. N. Engl. J. Med..

[13] Cuyvers E., De Roeck A., Van den Bossche T., Van Cauwenberghe C., Bettens K., Vermeulen S., Mattheijssens M., Peeters K., Engelborghs S., Vandenbulcke M. (2015). Mutations in ABCA7 in a Belgian cohort of Alzheimer’s disease patients: A targeted resequencing study. Lancet Neurol..

[14] Veteleanu A., Stevenson-Hoare J., Keat S., Daskoulidou N., Zetterberg H., Heslegrave A., Escott-Price V., Williams J., Sims R., Zelek W.M. (2023). Alzheimer’s disease-associated complement gene variants influence plasma complement protein levels. J. Neuroinflamm..

[15] Escott-Price V., Sims R., Bannister C., Harold D., Vronskaya M., Majounie E., Badarinarayan N., Perades G., Consortia I., Morgan K. (2015). Common polygenic variation enhances risk prediction for Alzheimer’s disease. Brain.

[16] Reiman E.M., Arboleda-Velasquez J.F., Quiroz Y.T., Huentelman M.J., Beach T.G., Caselli R.J., Chen Y., Su Y., Myers A.J., Hardy J. (2020). Exceptionally low likelihood of Alzheimer’s dementia in APOE2 homozygotes from a 5000-person neuropathological study. Nat. Commun..

[17] Verghese P.B., Castellano J.M., Holtzman D.M. (2011). Apolipoprotein E in Alzheimer’s disease and other neurological disorders. Lancet Neurol..

[18] Chen Y., Strickland M.R., Soranno A., Holtzman D.M. (2021). Apolipoprotein E: Structural Insights and Links to Alzheimer Disease Pathogenesis. Neuron.

[19] Jonsson T., Atwal J.K., Steinberg S., Snaedal J., Jonsson P.V., Bjornsson S., Stefansson H., Sulem P., Gudbjartsson D., Maloney J. (2012). A mutation in APP protects against Alzheimer’s disease and age-related cognitive decline. Nature.

[20] Sims R., van der Lee S.J., Naj A.C., Bellenguez C., Badarinarayan N., Jakobsdottir J., Kunkle B.W., Boland A., Raybould R., Bis J.C. (2017). Rare coding variants in PLCG2, ABI3, and TREM2 implicate microglial-mediated innate immunity in Alzheimer’s disease. Nat. Genet..

[21] Samuelsson J., Najar J., Wallengren O., Kern S., Wetterberg H., Mellqvist Fassberg M., Zetterberg H., Blennow K., Lissner L., Rothenberg E. (2022). Interactions between dietary patterns and genetic factors in relation to incident dementia among 70-year-olds. Eur. J. Nutr..

[22] Pedrero-Chamizo R., Zhuang K., Juarez A., Janabi M., Jagust W.J., Landau S.M. (2024). Alzheimer’s disease prevention: Apolipoprotein e4 moderates the effect of physical activity on brain beta-amyloid deposition in healthy older adults. J. Sci. Med. Sport.

[23] Querfurth H.W., LaFerla F.M. (2010). Alzheimer’s disease. N. Engl. J. Med..

[24] Wolfe M.S. (2021). Targeting gamma-Secretase for Familial Alzheimer’s Disease. Med. Chem. Res..

[25] Manescu M.D., Catalin B., Baldea I., Mateescu V.O., Rosu G.C., Boboc I.K.S., Istrate-Ofiteru A.M., Liliac I.M., Streba C.T., Kumar-Singh S. (2025). Aquaporin 4 modulation drives amyloid burden and cognitive abilities in an APPPS1 mouse model of Alzheimer’s disease. Alzheimers Dement.

[26] Andersson E., Lindblom N., Janelidze S., Salvado G., Gkanatsiou E., Soderberg L., Moller C., Lannfelt L., Ge J., Hanrieder J. (2025). Soluble cerebral Abeta protofibrils link Abeta plaque pathology to changes in CSF Aβ_42_/Aβ_40_ ratios, neurofilament light and tau in Alzheimer’s disease model mice. Nat. Aging.

[27] Almeida Z.L., Vaz D.C., Brito R.M.M. (2025). Morphological and Molecular Profiling of Amyloid-beta Species in Alzheimer’s Pathogenesis. Mol. Neurobiol..

[28] Huang Y., Rafael Guimaraes T., Todd N., Ferguson C., Weiss K.M., Stauffer F.R., McDermott B., Hurtle B.T., Saito T., Saido T.C. (2022). G protein-biased GPR3 signaling ameliorates amyloid pathology in a preclinical Alzheimer’s disease mouse model. Proc. Natl. Acad. Sci. USA.

[29] Holtzman D.M., Carrillo M.C., Hendrix J.A., Bain L.J., Catafau A.M., Gault L.M., Goedert M., Mandelkow E., Mandelkow E.M., Miller D.S. (2016). Tau: From research to clinical development. Alzheimers Dement.

[30] Ranasinghe K.G., Kudo K., Syed F., Yballa C., Kramer J.H., Miller B.L., Rankin K.P., Garcia P.A., Kirsch H.E., Vossel K. (2025). Distinct manifestations of excitatory-inhibitory imbalance associated with amyloid-beta and tau in patients with Alzheimer’s disease. Nat. Commun..

[31] Serrano-Pozo A., Frosch M.P., Masliah E., Hyman B.T. (2011). Neuropathological alterations in Alzheimer disease. Cold Spring Harb. Perspect. Med..

[32] Ossenkoppele R., van der Kant R., Hansson O. (2022). Tau biomarkers in Alzheimer’s disease: Towards implementation in clinical practice and trials. Lancet Neurol..

[33] Prusiner S.B. (2012). Cell biology. A unifying role for prions in neurodegenerative diseases. Science.

[34] Gibbons G.S., Lee V.M.Y., Trojanowski J.Q. (2019). Mechanisms of Cell-to-Cell Transmission of Pathological Tau: A Review. JAMA Neurol.

[35] Estus S., Shaw B.C., Devanney N., Katsumata Y., Press E.E., Fardo D.W. (2019). Evaluation of CD33 as a genetic risk factor for Alzheimer’s disease. Acta Neuropathol..

[36] Zhang L., Xiang X., Li Y., Bu G., Chen X.F. (2025). TREM2 and sTREM2 in Alzheimer’s disease: From mechanisms to therapies. Mol. Neurodegener..

[37] Nimmerjahn A., Kirchhoff F., Helmchen F. (2005). Resting microglial cells are highly dynamic surveillants of brain parenchyma in vivo. Science.

[38] Fruhwurth S., Zetterberg H., Paludan S.R. (2024). Microglia and amyloid plaque formation in Alzheimer’s disease—Evidence, possible mechanisms, and future challenges. J. Neuroimmunol..

[39] Salter M.W., Stevens B. (2017). Microglia emerge as central players in brain disease. Nat. Med..

[40] Heppner F.L., Ransohoff R.M., Becher B. (2015). Immune attack: The role of inflammation in Alzheimer disease. Nat. Rev. Neurosci..

[41] Ebrahimi R., Shahrokhi Nejad S., Falah Tafti M., Karimi Z., Sadr S.R., Ramadhan Hussein D., Talebian N., Esmaeilpour K. (2025). Microglial activation as a hallmark of neuroinflammation in Alzheimer’s disease. Metab. Brain Dis..

[42] Osborn L.M., Kamphuis W., Wadman W.J., Hol E.M. (2016). Astrogliosis: An integral player in the pathogenesis of Alzheimer’s disease. Prog. Neurobiol..

[43] Liddelow S.A., Barres B.A. (2017). Reactive Astrocytes: Production, Function, and Therapeutic Potential. Immunity.

[44] Govindpani K., McNamara L.G., Smith N.R., Vinnakota C., Waldvogel H.J., Faull R.L., Kwakowsky A. (2019). Vascular Dysfunction in Alzheimer’s Disease: A Prelude to the Pathological Process or a Consequence of It?. J. Clin. Med..

[45] O’Brien J.T., Erkinjuntti T., Reisberg B., Roman G., Sawada T., Pantoni L., Bowler J.V., Ballard C., DeCarli C., Gorelick P.B. (2003). Vascular cognitive impairment. Lancet Neurol..

[46] Montagne A., Nation D.A., Sagare A.P., Barisano G., Sweeney M.D., Chakhoyan A., Pachicano M., Joe E., Nelson A.R., D’Orazio L.M. (2020). APOE4 leads to blood-brain barrier dysfunction predicting cognitive decline. Nature.

[47] Nation D.A., Sweeney M.D., Montagne A., Sagare A.P., D’Orazio L.M., Pachicano M., Sepehrband F., Nelson A.R., Buennagel D.P., Harrington M.G. (2019). Blood-brain barrier breakdown is an early biomarker of human cognitive dysfunction. Nat. Med..

[48] Lee A.J., Raghavan N.S., Bhattarai P., Siddiqui T., Sariya S., Reyes-Dumeyer D., Flowers X.E., Cardoso S.A.L., De Jager P.L., Bennett D.A. (2022). FMNL2 regulates gliovascular interactions and is associated with vascular risk factors and cerebrovascular pathology in Alzheimer’s disease. Acta Neuropathol..

[49] Chen Y.H., Wang Z.B., Liu X.P., Mao Z.Q., Alzheimer’s Disease Neuroimaging Initiative (2025). Elevated serum sodium is linked to increased amyloid-dependent tau pathology, neurodegeneration, and cognitive impairment in Alzheimer’s disease. J. Neurochem..

[50] Erickson M.A., Johnson R.S., Damodarasamy M., MacCoss M.J., Keene C.D., Banks W.A., Reed M.J. (2024). Data-independent acquisition proteomic analysis of the brain microvasculature in Alzheimer’s disease identifies major pathways of dysfunction and upregulation of cytoprotective responses. Fluids Barriers CNS.

[51] Qiu J., Peng S., Qu R., Wu L., Xing L., Zhang L., Sun J. (2024). New evidence of vascular defects in neurodegenerative diseases revealed by single cell RNA sequencing. Clin. Sci..

[52] Nunomura A., Perry G., Aliev G., Hirai K., Takeda A., Balraj E.K., Jones P.K., Ghanbari H., Wataya T., Shimohama S. (2001). Oxidative damage is the earliest event in Alzheimer disease. J. Neuropathol. Exp. Neurol..

[53] Ahmed N., Ahmed U., Thornalley P.J., Hager K., Fleischer G., Münch G. (2005). Protein glycation, oxidation and nitration adduct residues and free adducts of cerebrospinal fluid in Alzheimer’s disease and link to cognitive impairment. J. Neurochem..

[54] Keller J.N., Schmitt F.A., Scheff S.W., Ding Q., Chen Q., Butterfield D.A., Markesbery W.R. (2005). Evidence of increased oxidative damage in subjects with mild cognitive impairment. Neurology.

[55] Roberts L.J., Montine T.J., Markesbery W.R., Tapper A.R., Hardy P., Chemtob S., Dettbarn W.D., Morrow J.D. (1998). Formation of isoprostane-like compounds (neuroprostanes) in vivo from docosahexaenoic acid. J. Biol. Chem..

[56] Leuzy A., Cullen N.C., Mattsson-Carlgren N., Hansson O. (2021). Current advances in plasma and cerebrospinal fluid biomarkers in Alzheimer’s disease. Curr. Opin. Neurol..

[57] Pereira J.B., Westman E., Hansson O., Alzheimer’s Disease Neuroimaging Initiative (2017). Association between cerebrospinal fluid and plasma neurodegeneration biomarkers with brain atrophy in Alzheimer’s disease. Neurobiol. Aging.

[58] Toledo J.B., Zetterberg H., van Harten A.C., Glodzik L., Martinez-Lage P., Bocchio-Chiavetto L., Rami L., Hansson O., Sperling R., Engelborghs S. (2015). Alzheimer’s disease cerebrospinal fluid biomarker in cognitively normal subjects. Brain.

[59] Kandimalla R.J., Prabhakar S., Wani W.Y., Kaushal A., Gupta N., Sharma D.R., Grover V.K., Bhardwaj N., Jain K., Gill K.D. (2013). CSF p-Tau levels in the prediction of Alzheimer’s disease. Biol. Open.

[60] Barthelemy N.R., Saef B., Li Y., Gordon B.A., He Y., Horie K., Stomrud E., Salvado G., Janelidze S., Sato C. (2023). CSF tau phosphorylation occupancies at T217 and T205 represent improved biomarkers of amyloid and tau pathology in Alzheimer’s disease. Nat. Aging.

[61] Khalafi M., Dartora W.J., McIntire L.B.J., Butler T.A., Wartchow K.M., Hojjati S.H., Razlighi Q.R., Shirbandi K., Zhou L., Chen K. (2025). Diagnostic accuracy of phosphorylated tau217 in detecting Alzheimer’s disease pathology among cognitively impaired and unimpaired: A systematic review and meta-analysis. Alzheimers Dement.

[62] Halbgebauer S., Steinacker P., Riedel D., Oeckl P., Anderl-Straub S., Lombardi J., von Arnim C.A.F., Nagl M., Giese A., Ludolph A.C. (2022). Visinin-like protein 1 levels in blood and CSF as emerging markers for Alzheimer’s and other neurodegenerative diseases. Alzheimers Res. Ther..

[63] Wang Y.Y., Zhang M., Chen S.J., Miao W., Wang Z.X., Zhou Y.J., Yu S.Q., Sun Z.W., Zhou X., Yu X.F. (2025). Neuroinflammation-mediated YKL-40 correlates with tau pathology and predicts longitudinal cognitive impairment and brain atrophy in Alzheimer’s disease, with hypertensive dependency. Front. Aging Neurosci..

[64] Wolner S.H., Gleerup H.S., Musaeus C.S., Hogh P., Ashton N.J., Brinkmalm A., Nilsson J., Grotschel L., Zetterberg H., Blennow K. (2025). Synaptosomal-Associated Protein 25 kDA (SNAP-25) Levels in Cerebrospinal Fluid: Implications for Alzheimer’s Disease Diagnosis and Monitoring. Synapse.

[65] Duits F.H., Brinkmalm G., Teunissen C.E., Brinkmalm A., Scheltens P., Van der Flier W.M., Zetterberg H., Blennow K. (2018). Synaptic proteins in CSF as potential novel biomarkers for prognosis in prodromal Alzheimer’s disease. Alzheimers Res. Ther..

[66] Galasko D., Xiao M., Xu D., Smirnov D., Salmon D.P., Dewit N., Vanbrabant J., Jacobs D., Vanderstichele H., Vanmechelen E. (2019). Synaptic biomarkers in CSF aid in diagnosis, correlate with cognition and predict progression in MCI and Alzheimer’s disease. Alzheimers Dement.

[67] Hoglund K., Schussler N., Kvartsberg H., Smailovic U., Brinkmalm G., Liman V., Becker B., Zetterberg H., Cedazo-Minguez A., Janelidze S. (2020). Cerebrospinal fluid neurogranin in an inducible mouse model of neurodegeneration: A translatable marker of synaptic degeneration. Neurobiol. Dis..

[68] Janelidze S., Hertze J., Zetterberg H., Landqvist Waldo M., Santillo A., Blennow K., Hansson O. (2016). Cerebrospinal fluid neurogranin and YKL-40 as biomarkers of Alzheimer’s disease. Ann. Clin. Transl. Neurol..

[69] Kim K.Y., Shin K.Y., Chang K.A. (2023). GFAP as a Potential Biomarker for Alzheimer’s Disease: A Systematic Review and Meta-Analysis. Cells.

[70] Kandimalla R.J., Anand R., Veeramanikandan R., Wani W.Y., Prabhakar S., Grover V.K., Bharadwaj N., Jain K., Gill K.D. (2014). CSF ubiquitin as a specific biomarker in Alzheimer’s disease. Curr. Alzheimer Res..

[71] Sjodin S., Hansson O., Ohrfelt A., Brinkmalm G., Zetterberg H., Brinkmalm A., Blennow K. (2017). Mass Spectrometric Analysis of Cerebrospinal Fluid Ubiquitin in Alzheimer’s Disease and Parkinsonian Disorders. Proteom. Clin. Appl..

[72] Du Y., Wu H.T., Qin X.Y., Cao C., Liu Y., Cao Z.Z., Cheng Y. (2018). Postmortem Brain, Cerebrospinal Fluid, and Blood Neurotrophic Factor Levels in Alzheimer’s Disease: A Systematic Review and Meta-Analysis. J. Mol. Neurosci..

[73] Frisoni G.B., Bocchetta M., Chetelat G., Rabinovici G.D., de Leon M.J., Kaye J., Reiman E.M., Scheltens P., Barkhof F., Black S.E. (2013). Imaging markers for Alzheimer disease: Which vs how. Neurology.

[74] Drzezga A., Barthel H. (2025). Imaging and Fluid Biomarkers of Alzheimer Disease: Complementation Rather Than Competition. J. Nucl. Med..

[75] Rabinovici G.D., Gatsonis C., Apgar C., Chaudhary K., Gareen I., Hanna L., Hendrix J., Hillner B.E., Olson C., Lesman-Segev O.H. (2019). Association of Amyloid Positron Emission Tomography With Subsequent Change in Clinical Management Among Medicare Beneficiaries With Mild Cognitive Impairment or Dementia. JAMA.

[76] Anand K., Sabbagh M. (2017). Amyloid Imaging: Poised for Integration into Medical Practice. Neurotherapeutics.

[77] Guerra U.P., Nobili F.M., Padovani A., Perani D., Pupi A., Sorbi S., Trabucchi M. (2015). Recommendations from the Italian Interdisciplinary Working Group (AIMN, AIP, SINDEM) for the utilization of amyloid imaging in clinical practice. Neurol. Sci..

[78] Laforce R., Rosa-Neto P., Soucy J.P., Rabinovici G.D., Dubois B., Gauthier S. (2016). Canadian Consensus Guidelines on Use of Amyloid Imaging in Canada: Update and Future Directions from the Specialized Task Force on Amyloid imaging in Canada. Can. J. Neurol. Sci..

[79] Scheltens P., De Strooper B., Kivipelto M., Holstege H., Chetelat G., Teunissen C.E., Cummings J., van der Flier W.M. (2021). Alzheimer’s disease. Lancet.

[80] Chen M.K., Mecca A.P., Naganawa M., Finnema S.J., Toyonaga T., Lin S.F., Najafzadeh S., Ropchan J., Lu Y., McDonald J.W. (2018). Assessing Synaptic Density in Alzheimer Disease With Synaptic Vesicle Glycoprotein 2A Positron Emission Tomographic Imaging. JAMA Neurol..

[81] Leuzy A., Chiotis K., Lemoine L., Gillberg P.G., Almkvist O., Rodriguez-Vieitez E., Nordberg A. (2019). Tau PET imaging in neurodegenerative tauopathies-still a challenge. Mol. Psychiatry.

[82] Zhang Q., Lu J., Wang L., Jiao F., Wang M., Shi K., Zuo C., Jiang J. (2025). Evaluating ^18^F-Florzolotau tau PET for Alzheimer’s disease diagnosis with ^18^F-Flortaucipir as reference. J. Neurol..

[83] Ossenkoppele R., Coomans E.M., Apostolova L.G., Baker S.L., Barthel H., Beach T.G., Benzinger T.L.S., Betthauser T., Bischof G.N., Bottlaender M. (2025). Tau PET positivity in individuals with and without cognitive impairment varies with age, amyloid-beta status, APOE genotype and sex. Nat. Neurosci..

[84] Liebsch F., Kulic L., Teunissen C., Shobo A., Ulku I., Engelschalt V., Hancock M.A., van der Flier W.M., Kunach P., Rosa-Neto P. (2019). Aβ_34_ is a BACE1-derived degradation intermediate associated with amyloid clearance and Alzheimer’s disease progression. Nat. Commun..

[85] Mattsson N., Cullen N.C., Andreasson U., Zetterberg H., Blennow K. (2019). Association Between Longitudinal Plasma Neurofilament Light and Neurodegeneration in Patients With Alzheimer Disease. JAMA Neurol..

[86] Spitzer P., Mulzer L.M., Oberstein T.J., Munoz L.E., Lewczuk P., Kornhuber J., Herrmann M., Maler J.M. (2019). Microvesicles from cerebrospinal fluid of patients with Alzheimer’s disease display reduced concentrations of tau and APP protein. Sci. Rep..

[87] Cai Z., Li S., Matuskey D., Nabulsi N., Huang Y. (2019). PET imaging of synaptic density: A new tool for investigation of neuropsychiatric diseases. Neurosci. Lett..

[88] Davies P., Maloney A.J. (1976). Selective loss of central cholinergic neurons in Alzheimer’s disease. Lancet.

[89] Li D.D., Zhang Y.H., Zhang W., Zhao P. (2019). Meta-Analysis of Randomized Controlled Trials on the Efficacy and Safety of Donepezil, Galantamine, Rivastigmine, and Memantine for the Treatment of Alzheimer’s Disease. Front Neurosci..

[90] Howard R., McShane R., Lindesay J., Ritchie C., Baldwin A., Barber R., Burns A., Dening T., Findlay D., Holmes C. (2012). Donepezil and memantine for moderate-to-severe Alzheimer’s disease. N. Engl. J. Med..

[91] McShane R., Westby M.J., Roberts E., Minakaran N., Schneider L., Farrimond L.E., Maayan N., Ware J., Debarros J. (2019). Memantine for dementia. Cochrane Database Syst. Rev..

[92] Mitani Y., Yarimizu J., Saita K., Uchino H., Akashiba H., Shitaka Y., Ni K., Matsuoka N. (2012). Differential effects between gamma-secretase inhibitors and modulators on cognitive function in amyloid precursor protein-transgenic and nontransgenic mice. J. Neurosci..

[93] May P.C., Dean R.A., Lowe S.L., Martenyi F., Sheehan S.M., Boggs L.N., Monk S.A., Mathes B.M., Mergott D.J., Watson B.M. (2011). Robust central reduction of amyloid-beta in humans with an orally available, non-peptidic beta-secretase inhibitor. J. Neurosci..

[94] Yao J., Du H., Yan S., Fang F., Wang C., Lue L.F., Guo L., Chen D., Stern D.M., Gunn Moore F.J. (2011). Inhibition of amyloid-beta (Abeta) peptide-binding alcohol dehydrogenase-Abeta interaction reduces Abeta accumulation and improves mitochondrial function in a mouse model of Alzheimer’s disease. J. Neurosci..

[95] Bachmeier C., Beaulieu-Abdelahad D., Mullan M., Paris D. (2013). Role of the cannabinoid system in the transit of beta-amyloid across the blood-brain barrier. Mol. Cell. Neurosci..

[96] Currais A., Quehenberger O., Armando A.M., Daugherty D., Maher P., Schubert D. (2016). Amyloid proteotoxicity initiates an inflammatory response blocked by cannabinoids. npj Aging Mech. Dis..

[97] Orgogozo J.M., Gilman S., Dartigues J.F., Laurent B., Puel M., Kirby L.C., Jouanny P., Dubois B., Eisner L., Flitman S. (2003). Subacute meningoencephalitis in a subset of patients with AD after Abeta42 immunization. Neurology.

[98] Vellas B., Black R., Thal L.J., Fox N.C., Daniels M., McLennan G., Tompkins C., Leibman C., Pomfret M., Grundman M. (2009). Long-term follow-up of patients immunized with AN1792: Reduced functional decline in antibody responders. Curr. Alzheimer Res..

[99] Winblad B., Andreasen N., Minthon L., Floesser A., Imbert G., Dumortier T., Maguire R.P., Blennow K., Lundmark J., Staufenbiel M. (2012). Safety, tolerability, and antibody response of active Abeta immunotherapy with CAD106 in patients with Alzheimer’s disease: Randomised, double-blind, placebo-controlled, first-in-human study. Lancet Neurol..

[100] Chai X., Wu S., Murray T.K., Kinley R., Cella C.V., Sims H., Buckner N., Hanmer J., Davies P., O’Neill M.J. (2011). Passive immunization with anti-Tau antibodies in two transgenic models: Reduction of Tau pathology and delay of disease progression. J. Biol. Chem..

[101] Salloway S., Sperling R., Fox N.C., Blennow K., Klunk W., Raskind M., Sabbagh M., Honig L.S., Porsteinsson A.P., Ferris S. (2014). Two phase 3 trials of bapineuzumab in mild-to-moderate Alzheimer’s disease. N. Engl. J. Med..

[102] Vandenberghe R., Rinne J.O., Boada M., Katayama S., Scheltens P., Vellas B., Tuchman M., Gass A., Fiebach J.B., Hill D. (2016). Bapineuzumab for mild to moderate Alzheimer’s disease in two global, randomized, phase 3 trials. Alzheimers Res. Ther..

[103] Doody R.S., Thomas R.G., Farlow M., Iwatsubo T., Vellas B., Joffe S., Kieburtz K., Raman R., Sun X., Aisen P.S. (2014). Phase 3 trials of solanezumab for mild-to-moderate Alzheimer’s disease. N. Engl. J. Med..

[104] Hatami A., Albay R., Monjazeb S., Milton S., Glabe C. (2014). Monoclonal antibodies against Abeta42 fibrils distinguish multiple aggregation state polymorphisms in vitro and in Alzheimer disease brain. J. Biol. Chem..

[105] Adolfsson O., Pihlgren M., Toni N., Varisco Y., Buccarello A.L., Antoniello K., Lohmann S., Piorkowska K., Gafner V., Atwal J.K. (2012). An effector-reduced anti-β-amyloid (Aβ) antibody with unique abeta binding properties promotes neuroprotection and glial engulfment of Abeta. J. Neurosci..

[106] Garber K. (2012). Genentech’s Alzheimer’s antibody trial to study disease prevention. Nat. Biotechnol..

[107] Jeon S., Bose S., Hur J., Jun K., Kim Y.K., Cho K.S., Koo B.S. (2011). A modified formulation of Chinese traditional medicine improves memory impairment and reduces Abeta level in the Tg-APPswe/PS1dE9 mouse model of Alzheimer’s disease. J. Ethnopharmacol..

[108] Ihl R., Tribanek M., Bachinskaya N., Group G.S. (2012). Efficacy and tolerability of a once daily formulation of Ginkgo biloba extract EGb 761(R) in Alzheimer’s disease and vascular dementia: Results from a randomised controlled trial. Pharmacopsychiatry.

[109] Jang S.I., Pae H.O., Choi B.M., Oh G.S., Jeong S., Lee H.J., Kim H.Y., Kang K.J., Yun Y.G., Kim Y.C. (2003). Salidroside from Rhodiola sachalinensis protects neuronal PC12 cells against cytotoxicity induced by amyloid-beta. Immunopharmacol. Immunotoxicol..

[110] Zhang L., Yu H., Zhao X., Lin X., Tan C., Cao G., Wang Z. (2010). Neuroprotective effects of salidroside against beta-amyloid-induced oxidative stress in SH-SY5Y human neuroblastoma cells. Neurochem. Int..

[111] Qu Z.Q., Zhou Y., Zeng Y.S., Lin Y.K., Li Y., Zhong Z.Q., Chan W.Y. (2012). Protective effects of a Rhodiola crenulata extract and salidroside on hippocampal neurogenesis against streptozotocin-induced neural injury in the rat. PLoS ONE.

[112] Cicero A.F.G., Fogacci F., Banach M. (2018). Botanicals and phytochemicals active on cognitive decline: The clinical evidence. Pharmacol. Res..

[113] Yang F., Lim G.P., Begum A.N., Ubeda O.J., Simmons M.R., Ambegaokar S.S., Chen P.P., Kayed R., Glabe C.G., Frautschy S.A. (2005). Curcumin inhibits formation of amyloid beta oligomers and fibrils, binds plaques, and reduces amyloid in vivo. J. Biol. Chem..

[114] Lou S., Gong D., Yang M., Qiu Q., Luo J., Chen T. (2024). Curcumin Improves Neurogenesis in Alzheimer’s Disease Mice via the Upregulation of Wnt/beta-Catenin and BDNF. Int. J. Mol. Sci..

[115] Panickar K.S., Qin B., Anderson R.A. (2015). Ischemia-induced endothelial cell swelling and mitochondrial dysfunction are attenuated by cinnamtannin D1, green tea extract, and resveratrol in vitro. Nutr. Neurosci..

[116] Manczak M., Mao P., Calkins M.J., Cornea A., Reddy A.P., Murphy M.P., Szeto H.H., Park B., Reddy P.H. (2010). Mitochondria-targeted antioxidants protect against amyloid-beta toxicity in Alzheimer’s disease neurons. J. Alzheimers Dis..

[117] Galasko D.R., Peskind E., Clark C.M., Quinn J.F., Ringman J.M., Jicha G.A., Cotman C., Cottrell B., Montine T.J., Thomas R.G. (2012). Antioxidants for Alzheimer disease: A randomized clinical trial with cerebrospinal fluid biomarker measures. Arch. Neurol..

[118] Puranik N., Kumari M., Tiwari S., Dhakal T., Song M. (2025). Resveratrol as a Therapeutic Agent in Alzheimer’s Disease: Evidence from Clinical Studies. Nutrients.

[119] Dysken M.W., Sano M., Asthana S., Vertrees J.E., Pallaki M., Llorente M., Love S., Schellenberg G.D., McCarten J.R., Malphurs J. (2014). Effect of vitamin E and memantine on functional decline in Alzheimer disease: The TEAM-AD VA cooperative randomized trial. JAMA.

[120] Gong Q.H., Wang Q., Pan L.L., Liu X.H., Xin H., Zhu Y.Z. (2011). S-propargyl-cysteine, a novel hydrogen sulfide-modulated agent, attenuates lipopolysaccharide-induced spatial learning and memory impairment: Involvement of TNF signaling and NF-kappaB pathway in rats. Brain. Behav. Immun..

[121] Laxton A.W., Tang-Wai D.F., McAndrews M.P., Zumsteg D., Wennberg R., Keren R., Wherrett J., Naglie G., Hamani C., Smith G.S. (2010). A phase I trial of deep brain stimulation of memory circuits in Alzheimer’s disease. Ann. Neurol..

[122] Leinenga G., Gotz J. (2015). Scanning ultrasound removes amyloid-beta and restores memory in an Alzheimer’s disease mouse model. Sci. Transl. Med..

[123] Leinenga G., To X.V., Bodea L.G., Yousef J., Richter-Stretton G., Palliyaguru T., Chicoteau A., Dagley L., Nasrallah F., Gotz J. (2024). Scanning ultrasound-mediated memory and functional improvements do not require amyloid-beta reduction. Mol. Psychiatry.

[124] De la Rosa A., Olaso-Gonzalez G., Arc-Chagnaud C., Millan F., Salvador-Pascual A., Garcia-Lucerga C., Blasco-Lafarga C., Garcia-Dominguez E., Carretero A., Correas A.G. (2020). Physical exercise in the prevention and treatment of Alzheimer’s disease. J. Sport Health Sci..

[125] Erickson K.I., Voss M.W., Prakash R.S., Basak C., Szabo A., Chaddock L., Kim J.S., Heo S., Alves H., White S.M. (2011). Exercise training increases size of hippocampus and improves memory. Proc. Natl. Acad. Sci. USA.

[126] Northey J.M., Cherbuin N., Pumpa K.L., Smee D.J., Rattray B. (2018). Exercise interventions for cognitive function in adults older than 50: A systematic review with meta-analysis. Br. J. Sports Med..

[127] Li X., Jin Y., Ding X., Zhu T., Wei C., Yao L. (2023). Long-term exercise training inhibits inflammation by suppressing hippocampal NLRP3 in APP/PS1 mice. Sports Med. Health Sci..

[128] Liu W., Zhang J., Wang Y., Li J., Chang J., Jia Q. (2022). Effect of Physical Exercise on Cognitive Function of Alzheimer’s Disease Patients: A Systematic Review and Meta-Analysis of Randomized Controlled Trial. Front. Psychiatry.

[129] Tari A.R., Walker T.L., Huuha A.M., Sando S.B., Wisloff U. (2025). Neuroprotective mechanisms of exercise and the importance of fitness for healthy brain ageing. Lancet.

[130] Barnes L.L., Dhana K., Liu X., Carey V.J., Ventrelle J., Johnson K., Hollings C.S., Bishop L., Laranjo N., Stubbs B.J. (2023). Trial of the MIND Diet for Prevention of Cognitive Decline in Older Persons. N. Engl. J. Med..

[131] Agarwal P., Barnes L.L., Dhana K., Liu X., Zhang Y., Beck T., Cornelis M.C., Tangney C., Rajan K.B. (2024). Association of MIND diet with cognitive decline among Black and White older adults. Alzheimers Dement.

[132] Welty F.K. (2023). Omega-3 fatty acids and cognitive function. Curr. Opin. Lipidol..

[133] Patel S., Thornton A., Parmar M.S. (2025). Resveratrol’s Multifaceted Potential in Alzheimer’s Disease: Insights from Preclinical and Clinical Evidence. Mol. Neurobiol..

[134] Sowmiya S., Dhivya L.S., Harikrishnan N., Ankul Singh S. (2024). Exploring the potential of probiotics in Alzheimer’s disease and gut dysbiosis. IBRO Neurosci. Rep..

[135] Wang X., Sun G., Feng T., Zhang J., Huang X., Wang T., Xie Z., Chu X., Yang J., Wang H. (2019). Sodium oligomannate therapeutically remodels gut microbiota and suppresses gut bacterial amino acids-shaped neuroinflammation to inhibit Alzheimer’s disease progression. Cell Res..

[136] Bosch M.E., Dodiya H.B., Michalkiewicz J., Lee C., Shaik S.M., Weigle I.Q., Zhang C., Osborn J., Nambiar A., Patel P. (2024). Sodium oligomannate alters gut microbiota, reduces cerebral amyloidosis and reactive microglia in a sex-specific manner. Mol. Neurodegener..

[137] Ornish D., Madison C., Kivipelto M., Kemp C., McCulloch C.E., Galasko D., Artz J., Rentz D., Lin J., Norman K. (2024). Effects of intensive lifestyle changes on the progression of mild cognitive impairment or early dementia due to Alzheimer’s disease: A randomized, controlled clinical trial. Alzheimers Res. Ther..

